# Procedural Outcomes of a Self-Expanding Transcatheter Heart Valve in Small Annuli

**DOI:** 10.3390/jcm11185313

**Published:** 2022-09-09

**Authors:** Clemens Eckel, Dagmar Sötemann, Won-Keun Kim, Christina Grothusen, Vedat Tiyerili, Guido Dohmen, Matthias Renker, Efstratios Charitos, Christian W. Hamm, Yeong-Hoon Choi, Helge Möllmann, Johannes Blumenstein

**Affiliations:** 1 Department of Cardiology, St. Johannes Hospital, 44137 Dortmund, Germany; 2 Department of Cardiology, University of Oldenburg, 26129 Oldenburg, Germany; 3 Department of Cardiology, Kerckhoff Heart Center, 61231 Bad Nauheim, Germany; 4 Department of Cardiac Surgery, Kerckhoff Heart Center, 61231 Bad Nauheim, Germany; 5 Department of Cardiac Surgery, St. Johannes Hospital, 44137 Dortmund, Germany; 6 Department of Internal Medicine, St. Johannes Hospital, Johannesstraße 9-13, 44139 Dortmund, Germany

**Keywords:** TAVR, self-expanding, THV, ACCURATE, PPM, small annulus

## Abstract

Background: Self-expanding transcatheter valves (THV) seem superior to balloon-expanding valves in regard to the incidence of prosthesis–patient mismatch (PPM). Data on the occurrence of PPM with the ACURATE *neo/neo2* system as a representative of self-expanding prostheses in very small annuli, even below the applicable instructions for use (IFU), are scarce. Methods: Data from 654 patients with severe native aortic stenosis treated with the smallest size ACURATE *neo/neo2* valve (size S, 23 mm) at two German high-volume centers from 06/2012 to 12/2021 were evaluated. We compared clinical and hemodynamic outcomes among patients with implantation in adherence to the recommended sizing (on-label *n* = 529) and below the recommended sizing range (off-label *n* = 125) and identified predictors for PPM in the overall population. BMI-adjusted PPM was defined according to VARC-3 recommendations. Results: Post-procedure, the mean gradient (10.0 mmHg vs. 9.0 mmHg, *p* = 0.834) and the rate of paravalvular leakage (PVL) ≥ moderate (3.2% vs. 2.8%, *p* = 0.770) were similar between on-label and off-label implantations. The rate of moderate to severe PPM (24%) was comparably low in ACURATE *neo/neo2* S, with a very low proportion of severe PPM whether implanted off- or on-label (4.9% vs. 3.8%, *p* = 0.552). Thirty-day all-cause mortality was higher among patients with off-label implantations (6.5% vs. 2.3%, *p* = 0.036). In the subgroup of these patients, no device-related deaths occurred, and cardiac causes did not differ (each 5). Besides small annulus area and high BMI, a multivariate analysis identified a greater cover index (OR 3.26), deep implantation (OR 2.25) and severe calcification (OR 2.07) as independent predictors of PPM. Conclusions: The ACURATE *neo/neo2* S subgroup shows a convincing hemodynamic outcome according to low mean gradient even outside the previous IFUs without a relevant increase in the rate of PVL or PPM. In addition to known factors such as annulus area and BMI, potential predictors for PPM are severe annulus calcification and implantation depth. Nevertheless, the ACURATE *neo/neo2* system seems to be a reliable option in patients with very small annuli.

## 1. Introduction

Transcatheter aortic valve replacement (TAVR) has continued to evolve in recent years in terms of experience, technology and clinical application [1,2,3]. Significant differences have emerged between self-expanding and balloon-expanding prostheses, and the new generation of transcatheter heart valves (THVs) continues to address specific drawbacks such as the implantation of pacemakers or paravalvular leakage (PVL) [4,5]. Previous studies in populations with small annuli have mainly highlighted the risk of PPM and negative hemodynamic outcomes due to the narrow anatomy. Strategies to prevent prosthesis–patient mismatch (PPM) include the use of self-expanding supra-annular prostheses in TAVR or aortic root enlargement/replacement and the use of sutureless bioprostheses in surgical valve replacement (SAVR). This study investigates whether the favorable hemodynamic properties of the supra-annular ACURATE *neo/neo2* system provides feasibility in a subset of patients with small annuli even below the applicable instructions for use (IFU).

## 2. Methods

In this retrospective analysis, patients with severe native aortic stenosis who underwent transfemoral TAVR with the ACURATE *neo* (*n* = 464) or ACURATE *neo2* (*n* = 191) valve (Boston Scientific, Marlborough, MA, USA) at two German high-volume centers (Kerckhoff Heart Center, Bad Nauheim; St. Johannes Hospital, Dortmund, Germany) between June 2012 and December 2021 were included. The valve design and the implantation technique have been described previously [6,7]. Baseline characteristics such as comorbidities, risk scores, echocardiography, MDCT and cardiac catheterization data were prospectively recorded in a dedicated database, as were procedural data and complications from each participating center. The pooling and review of all data was led by a single investigator, and discrepancies were resolved through direct communication with both centers. Follow-up data were collected at outpatient visits, from recent medical reports or by telephone interview. The study was conducted according to the Declaration of Helsinki.

### 2.1. Multidetector Computed Tomography

Multidetector computed tomography (MDCT) was performed using a 64-slice or a 192-slice dual-source scanner (Somatom Definition or Somatom Force, Siemens Healthcare, Forchheim, Germany), as previously described [8]. For the analysis of MDCT datasets, a dedicated software was used (3mensio, Pie Medical, Maastricht, The Netherlands). Next to the standard measurements of aortic root dimensions, the cover index [CI = 100 × (prosthesis diameter − perimeter-derived annulus diameter)/prosthesis diameter (%)] and the relation between the sinotubular junction (STJ) and the perimeter-derived annulus was calculated as the STJ-annulus index [= 100 × (STJ − perimeter-derived annulus)/STJ (%)]. The aortic valve calcium score (AVCS) was measured according to the Agatston method using non-contrast-enhanced MDCT scans [9]. The calcium density (Ca-density) was calculated as AVCS/annular area (AU/cm^2^) [10]. The presence of eccentric aortic valve (AV) calcification and relevant left ventricular outflow tract (LVOT) calcification was determined by a visual estimation of the aortic valve in short axis views and maximum intensity projections, as previously described [11].

### 2.2. Assessment of PPM and Implantation Depth

PPM was defined according to the Valvular Academic Research Consortium (VARC)-3 criteria [12] as BMI adapted (iAVA if BMI < 30 kg/m^2^: none > 0.85 cm^2^/m^2^; moderate 0.85–0.66 cm^2^/m^2^; severe ≤ 0.66 cm^2^/m^2^; and iAVA if BMI ≥ 30 kg/m^2^: none > 0.70 cm^2^/m^2^; moderate 0.70–0.56 cm^2^/m^2^; severe ≤ 0.55 cm^2^/m^2^). PVL was assessed at discharge echocardiography using a three-class grading scheme (none/trace, mild, moderate, severe) in adherence to existing recommendations [12]. The implantation depth of the prosthesis was determined upon a final angiography at the non-coronary cusp (NCC) and the left coronary cusp (LCC), as described previously [13].

### 2.3. Outcomes of Interest

The primary outcome measure was echocardiographic performance described by post-interventional gradients, indicated aortic valve area (iAVA), PVL and possible prosthesis–patient mismatch (PPM), as described above. Secondary outcome measures were 30-day all-cause mortality, technical success, device success at 30 days, and the early safety combined endpoint at 30 days according to the recent VARC-3 document [12].

### 2.4. Statistical Analysis

The population was divided into two subsets according to whether the implantation was performed in line with the official recommendations of the manufacturer (on-label sizing) or below (off-label sizing). Continuous data are given as median and interquartile range [IQR]. The comparison of groups was accomplished using the Mann–Whitney U test and Fisher’s two-tailed exact test or the chi-square test, as indicated. Univariable logistic regression was used to determine the predictors of PPM, including the following variables: age, coronary artery disease, annulus area, CI for STJ, BMI, implantation depth, LVOT calcification, mean gradient, severe aortic valve calcification, post-dilatation, and ejection fraction. All variables with *p*-values < 0.1 in the univariate analysis were included in the multivariable analysis and were dichotomized if not already described as such, except for BMI and annulus area (defining target variable). For all analyses, a two-sided *p*-value < 0.05 was considered significant. All analyses were conducted using R version 4.2.1 (R Core Team (2021). R: A language and environment for statistical computing. R Foundation for Statistical Computing, Vienna, Austria. URL https://www.R-project.org/ (accessed on 4 February 2022)).

## 3. Results

### 3.1. Baseline Data

The mean age was 82.0 [79.0; 85.5] years and 93.3% were female. In terms of sizing, 125 patients (19.1%) had smaller annulus dimensions that were below the official recommendations (off-label sizing), whereas 529 patients (80.9%) were implanted in adherence to the official recommendations of the manufacturer (on-label sizing). There were no differences with respect to baseline parameters and recorded comorbidities, regardless of whether they received an ACURATE *neo/neo2* S with on-label or off-label sizing—see Table 1. Corresponding to the smaller annulus, the anatomical parameters differed for the derived annulus diameter (20.4 mm vs. 22.0 mm, *p* < 0.001), derived LVOT diameter (19.4 mm vs. 21.2 mm, *p* < 0.001) and derived STJ diameter (24.9 mm vs. 25.9 mm, *p* < 0.001).

### 3.2. Procedural Data and Outcomes 

The THV cover index at the annulus level was shown to be greater when implanted off-label (10.9% vs. 4.5%, *p* < 0.001). The rate of pre-dilation (58.4% vs. 72.8%, *p* = 0.002) as well as post-dilation (16.8% vs. 26.4%, *p* = 0.033) was lower when implanted off-label. Implantation depths at the NCC (6.0 mm vs. 6.0 mm; *p* = 0.966) and LCC (6.0 mm vs. 6.0 mm; *p* = 0.665) were equal. Technical success (88.8% vs. 90.5%; *p* = 0.671) and device success at 30 days (80.0% vs. 83.9%, *p* = 0.355) were comparable in both groups.

Periprocedural complications according to VARC-3 criteria were comparable in both groups. Bleeding type 2–4 was comparable without a difference between the groups (22.6% vs. 21.9%, *p* = 0.970)—see Table 2. Major cardiac structural complications were rare (1.6% vs. 1.5%, *p* = 1.000). There was no difference of patients with overt CNS injury (4.0% vs. 2.8%, *p* = 0.561) or acute kidney injury stage 2–4 (6.4% vs. 4.5%, *p* = 0.524). The 30-day all-cause mortality showed a higher rate when implanted off-label (6.5% vs. 2.3%, *p* = 0.036) with an increased rate of in-hospital death (5.6% vs. 1.7%, *p* = 0.020). A close look at the cause of death (see Table 3) shows a distribution between inflammatory/septic, cardiac or procedural complications (each 5), thromboembolic and multiorgan failure (MOF), with no significant difference between the groups (*p* < 0.900). No device-related deaths occurred.

### 3.3. Hemodynamics and PPM

**Figure 1:**
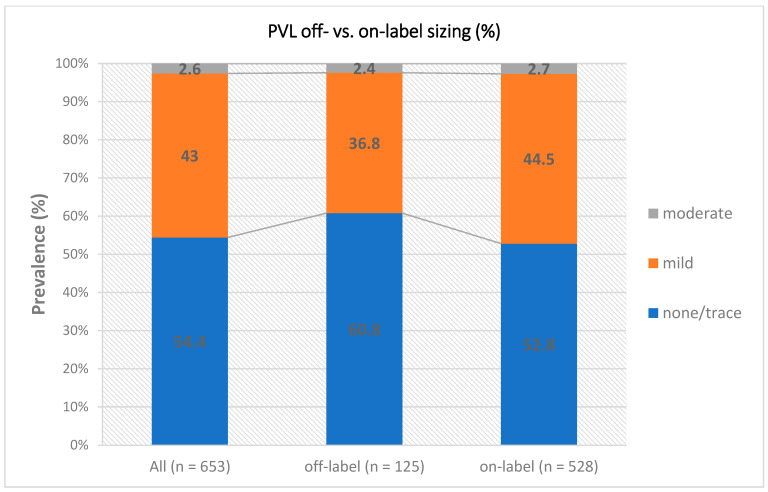
PVL on- vs. off-label sizing. Abbreviation: PVL = paravalvular leak.

Transthoracic echocardiography assessment before discharge showed low mean transprosthetic gradients (10.0 mmHg vs. 9.0 mmHg, *p* = 0.834) and low rates of more than mild PVL (3.2% vs. 2.8%, *p* = 0.770) in the collective of small annuli (see Figure 1). A significantly smaller aortic valve area was seen in relation to body surface area (0.87 cm^2^/m^2^ vs. 0.92 cm^2^/m^2^, *p* = 0.029). Numerically, there was a tendency towards an increased rate of moderate and severe PPM (35.4% vs. 26.5%, *p* = 0.113). Table 4 shows procedural parameters according to the occurrence of a PPM after implantation. Table 5 shows the multivariable logistic regression model for dichotomized parameters for the total population. In the overall cohort, lower annulus area, higher BMI, deeper implantation and severe calcification were independent predictors of moderate to severe PPM.

## 4. Discussion

Transcatheter aortic valve implantation has become the gold standard for treating most patients suffering from aortic valve stenosis. Nonetheless, there are still patients who do not fulfill classic sizing criteria, as indicated in the respective IFUs of different valve manufacturers. Within the current IFUs of the self-expanding THV systems, the ACURATE *neo/neo2* represents intermediate values with lower limits from a 21-mm diameter and 346-mm^2^ annulus area. In our collective, the ACURATE *neo/neo2* system was implanted off-label down to 18.7 mm and 260 mm^2^. The main findings of our study are: (1) ACURATE *neo/neo2* S showed favorable procedural results in small annuli (both within and below the IFU), low gradients and a low rate of severe PPM; (2) there is no significant difference in postprocedural gradients and the rate of PVL between on- and off-label implantation; (3) there is no difference in the rate of PPM when implanted off-label; (4) independent predictors of PPM were a greater cover index (STJ), a severely calcified annulus and deep implantation; (5) the 30-day all-cause mortality showed a higher rate when sized off-label.

### 4.1. Prosthesis–Patient Mismatch and PVL

In this high-risk population of patients with small annuli, the SMALL registry [14] for ACURATE *neo/neo2* system showed the most convincing results in terms of the incidence of moderate (6.4%) to severe (4.7%) PPM. Only Evolut Pro had lower rates of severe PPM (2.2%), but higher rates of moderate PPM (18.3%) [14]. The self-expanding ACURATE *neo/neo2* system is considered superior to intra-annular systems for its preferable hemodynamic features [15]. Due to this, supra-annular self-expanding TAVI systems are increasingly recommended to avoid severe PPM [16,17]. To date, risk factors for the occurrence of PPM are largely unexplored. Previous studies have shown an increased risk of moderate to severe PPM with small annulus, balloon-expanding systems, severe calcification of the aortic valve, reduced ejection fraction and younger age, whereas the risk seems to be reduced after post-dilatation [16,18]. We can confirm these results in our collective, except for the role of ejection fraction and post-dilatation. This may be due to either the overall strikingly lower rate of pre-dilatation (70%) compared to previous studies with this system [14], or the relatively higher radial force of the ACURATE *neo/neo2* S prosthesis. The low median gradient with 10 mmHg (6–12 mmHg) when implanted TAVR off-label should be highlighted.

Regardless of the PPM defined according to VARC-3 criteria on iAVA, the gradients show a low rate of de facto PPM in patients with normal left ventricular function. According to the estimated AVA the rate of PPM is 24%, but functionally and according to the transprosthetic gradients it is still significantly lower. Patients with small annuli appeared to benefit with respect to hemodynamic outcome in studies comparing TAVR with SAVR [16]. This makes the ACURATE *neo/neo2* system a real alternative in direct comparison to surgical aortic valve replacement with frequently necessary aortic root dilation.

### 4.2. Pacemaker

The overall rate of pacemaker implantation in this cohort was low with (8.0% vs. 7.2%, *p* = 0.901) in relation to the latest data [17]. Regardless, the ACURATE *neo/neo2* system is characterized by an only moderate radial force, especially compared to the Evolut system [19] or balloon-expandable prostheses [20]. With regard to the outcome-relevant long-term effects of right ventricular pacing, low pacing rates should be aimed for [21].

### 4.3. Procedural and 30-Day Outcome

Technical success was high overall in the ACURATE *neo/neo2* S collective (90.2%). As already mentioned, the comparison of previous studies in the collective of small annuli shows a very low overall rate of pre- (58.4% vs. 72.8%, *p* = 0.002) and post-dilatation (16.8% vs. 26.4%, *p* = 0.033) with a markedly lower rate when off-label sized. According to this, Pagnesi et al. highlighted in the NEOPRO multicenter study the safety and feasibility of ACCURATE neo implantation without predilatation, especially in low calcified patients [22]. The rate of bleeding, vascular complications and CNS injury according to the latest VARC-3 criteria also showed only numerical differences when implanted off-label. Nevertheless, the rate of all-cause mortality intrahospital and after 30 days showed inferiority (6.5% vs. 2.3%, *p* = 0.036). Given the indifference in PPM, this factor can surely be ruled out as a potential underlying reason. In fact, a close look at the cause of death shows similar distribution on inflammatory/septic, cardiac or procedural complications, thromboembolic and MOF between the groups with no significant accumulation of cardiac causes. In general, PVL and PPM have been identified as predictors of worse outcomes, but the role in the subset of patients with small annuli is so far unclear. However, the incidence of significant PVL, contrary to the rate of PPM, appears to be significantly lower in patients with small aortic annuli than in patients with larger aortic annuli.

Therefore, the statistical difference in mortality is most likely due to the small sample failure (*n* = 8 and *n* = 12, respectively). Large-scale randomized trials with long-term follow-up are needed to assess the effects of PPM and PVL on patients with aortic stenosis in the small aortic annuli collective.

### 4.4. Limitations

The present analysis is, of course, limited by its retrospective, non-randomized nature. The relatively long period over which the study was included also introduces bias due to learning curve effects and different procedural approaches (e.g., changes in pre/post dilatation strategies, single femoral access, radial access for pigtail catheter). There was no adverse event monitoring, and the imaging data were not analyzed by a core laboratory. LVOT calcification and eccentric AV calcification were assessed visually without further quantification. Echocardiographic data are missing in 1.5% (10/654) patients. The follow up at 30 days was incomplete in 1.2% (8/654). However, the data set represents real-world data from two high-volume centers on the largest data set to date in the small annuli subgroup for the ACURATE *neo/neo2* system, which helps to overcome the above-mentioned potential biases.

## 5. Conclusions

The ACURATE neo system shows a low rate of PVL and PPM when implanted in small annuli, even outside the official instructions for use. A severely calcified annulus and deep implantation should be considered as potential predictors for calculated PPM. The ACURATE neo system is convincing as a reliable option, especially in patients with very small annuli, when weighed against surgical aortic valve replacement.

## Figures and Tables

**Table 1:** Baseline characteristics.

Variable	Off-Label Sizing	On-Label Sizing	*p* Value
	*n* = 125	*n* = 529	
Age, years	82.0 [79.9; 85.5]	82.0 [79.0; 85.6]	0.677
Female gender	119 (95.2%)	495 (93.6%)	0.635
BMI, kg/m^2^	26.6 [22.8; 30.1]	25.8 [23.4; 29.8]	0.928
EuroScore I, %	16.3 [10.7; 25.7]	16.7 [10.1; 24.3]	0.489
EuroScore II, %	3.3 [2.5; 4.9]	3.4 [2.3; 5.0]	0.931
eGFR, mL/min/1,73 m^2^	59.0 [42.0; 74.0]	55.0 [42.0; 75.0]	0.526
Peripheral artery disease	13 (10.4%)	60 (11.3%)	0.886
Prior stroke	8 (6.4%)	65 (12.3%)	0.085
Atrial fibrillation	43 (34.4%)	170 (32.1%)	0.704
Coronary artery disease	61 (48.8%)	303 (57.3%)	0.106
**Electrocardiographic data**			
Previous right bundle branch block	10 (8.0%)	43 (8.1%)	1.000
Previous left bundle branch block	8 (6.4%)	39 (7.4%)	0.852
Previous atrioventricular block	11 (8.8%)	76 (14.4%)	0.133
**Echocardiographic data**			
Ejection fraction, %	65.0 [60.0; 65.0]	65.0 [60.0; 65.0]	0.666
Mean gradient, mmHg	43.0 [34.0; 49.0]	43.0 [35.0; 54.0]	0.173
AVA, cm^2^	0.7 [0.6; 0.8]	0.7 [0.6; 0.8]	0.080
**MDCT data**			
Perimeter derived annulus diameter, mm	20.4 [20.1; 20.7]	22.0 [21.6;22.4]	<0.001
LVOT, mm	19.4 [18.1; 20.2]	21.2 [20.1;22.2]	<0.001
STJ, mm	24.9 [23.3; 26.5]	25.9 [24.7;27.1]	<0.001
Aortic valve calcification, AU	1372.0 [875.0; 2042.0]	1670.0 [1088.0; 2486.8]	0.001
Calcium density, AU/m^2^	427.7 [276.7; 684.1]	481.9 [317.4;732.1]	0.050
Calcification in LVOT	5 (4.0%)	24 (4.5%)	0.983
Eccentric calcification	11 (8.8%)	75 (14.2%)	0.146

Abbreviation: BMI = body mass index; eGFR = glomerular filtration rate; AVA = aortic valve area; LVOT = left ventricular outflow tract; STJ = sinotubular junction.

**Table 2:** Procedural outcomes and complications.

Variable	Off-Label Sizing	On-Label Sizing	*p* Value
	*n* = 125	*n* = 529	
**Procedural parameter**			
THV cover index (perimeter), %	10.9 [9.9; 12.6]	4.5 [2.6; 6.3]	<0.001
Procedural duration, min	47.0 [35.0; 61.0]	45.0 [36.0; 60.0]	0.691
Contrast agent, mL	80.0 [55.0; 106.0]	80.0 [55.8; 110.0]	0.627
Pre-dilatation, %	73 (58.4%)	385 (72.8%)	0.002
Post-dilatation, %	21 (16.8%)	138 (26.4%)	0.033
Depth NCC, mm	6.0 [4.0; 6.5]	6.0 [4.0; 6.5]	0.966
Depth LCC, mm	6.0 [5.0; 7.0]	6.0 [4.0; 7.0]	0.665
**Echocardiographic outcome**			
Ejection fraction, %	65.0 [63.0;65.0]	65.0 [60.0;65.0]	0.873
Mean gradient, mmHg	10.0 [6.0;12.0]	9.0 [6.8;13.0]	0.834
AVA, cm^2^	1.5 [1.3; 1.7]	1.6 [1.4;1.8]	<0.001
iAVA, cm^2^/m^2^	0.87 [0.7; 1.0]	0.92 [0.8;1.1]	0.029
**Clinical outcome**			
Technical success	111 (88.8%)	479 (90.5%)	0.671
Device success at 30 days	100 (80.0%)	444 (83.9%)	0.355
Early safety at 30 days	51 (40.8%)	220 (41.6%)	0.952
In-hospital death	7 (5.6%)	9 (1.7%)	0.020
All-cause death at 30 days	8 (6.5%)	12 (2.3%)	0.036
Relevant PVL (>mild/trace)	4 (3.2%)	15 (2.8%)	0.770
Moderate to severe PPM	29 (35.4%)	111 (26.5%)	0.113
Severe PPM	4 (4.9%)	16 (3.8%)	0.552
Conversion to sternotomy	2 (1.6%)	3 (0.6%)	0.244
Multiple valves (ViV)	2 (1.6%)	3 (0.6%)	0.244
Device embolization	4 (3.2%)	7 (1.3%)	0.235
Major vascular complication	12 (9.6%)	46 (8.7%)	0.885
Bleeding (type 2–4)	28 (22.6%)	116 (21.9%)	0.970
Overt CNS injury	5 (4.0%)	15 (2.8%)	0.561
Cardiac structural complication	2 (1.6%)	8 (1.5%)	1.000
AKI (type 2–4)	8 (6.4%)	24 (4.5%)	0.524
New permanent pacemaker ^1^	10 (8.4%)	38 (8.1%)	1.000

Abbreviation: THV = transcatheter heart valve; LCC = left coronary cusp; NCC = non coronary cusp; AVA = aortic valve area; iAVA = indexed aortic valve area; PVL = paravalvular leak; CNS = central nervous system; PPM = prosthesis–patient mismatch; AKI = acute kidney injury. ^1^ Excluded patients with preexisting pacemaker (*n* = 65) at baseline.

**Table 3:** Reason for death up to 30 days.

	Off-Label Sizing	On-Label Sizing	*p* Value
	*n* = 8	*n* = 12	
**All-cause death**			0.900
Cardiac/procedural related	5 (62.5%)	5 (41.7%)	
Inflammatory/sepsis	2 (25.0%)	3 (25.0%)	
Multiorgan failure (MOF)	1 (12.5%)	2 (16.7%)	
Stroke	0 (0.0%)	2 (16.7%)	

**Table 4:** Procedural data and outcome according to PPM.

	no PPM	PPM	*p* Value
	*n* = 361	*n* = 140	
**Procedural parameter**			
THV cover index (perimeter), %	5.0 [3.1; 7.5]	4.7 [3.1; 8.2]	0.637
THV cover index (STJ), %	−0.2 [−9.1; −0.1]	−0.1 [−0.2; −0.1]	**<0.001**
Procedural duration, min	45.0 [35.0; 58.0]	40.0 [33.0; 48.0]	**0.001**
Contrast agent, mL	77.0 [48.8; 104.2]	74.0 [50.0; 100.0]	0.647
Pre-dilatation, %	239 (66.2%)	94 (67.1%)	0.925
Post-dilatation, %	96 (26.9%)	32 (22.9%)	0.417
Depth NCC, mm	5.7 [4.0; 6.5]	6.0 [5.0; 6.5]	0.232
Depth LCC, mm	6.0 [4.0; 7.0]	6.0 [5.0; 7.0]	0.527
**Echocardiographic outcome**			
Ejection fraction, %	65.0 [61.0; 65.0]	65.0 [65.0; 65.0]	**0.092**
Mean gradient, mmHg	9.0 [6.0; 12.0]	13.0 [9.0; 15.0]	**<0.001**
AVA, cm^2^	1.7 [1.5; 1.8]	1.3 [1.2; 1.4]	**<0.001**
**Clinical outcome (VARC 3)**			
Technical success	338 (93.6%)	131 (93.6%)	1.000
Device success at 30 days	324 (89.8%)	114 (81.4%)	**0.018**
Early safety at 30 days	150 (41.6%)	37 (26.4%)	**0.002**
All-cause death at 30 days	2 (0.6%)	1 (0.7%)	1.000
In-hospital death	1 (0.3%)	1 (0.7%)	0.481
Relevant PVL	11 (3.0%)	5 (3.6%)	0.780
Conversion to sternotomy	2 (0.6%)	0 (0.0%)	1.000
Multiple valves (ViV)	1 (0.3%)	3 (2.1%)	0.068
Embolization	4 (1.1%)	3 (2.1%)	0.405
Major vascular complication	27 (7.5%)	10 (7.1%)	1.000
Bleed (type 2–4)	70 (19.4%)	23 (16.5%)	0.546
Overt CNS injury	7 (1.9%)	3 (2.1%)	1.000
AKI (type 2–4)	16 (4.4%)	3 (2.1%)	0.346
New permanent pacemaker ^1^	27 (7.5%)	7 (5.0%)	0.428

Abbreviation: THV = transcatheter heart valve; LCC = left coronary cusp; NCC = non coronary cusp; AVA = aortic valve area; iAVA = aortic valve area; PVL = paravalvular leak; CNS = central nervous system; PPM = prosthesis–patient mismatch; AKI = acute kidney injury. ^1^ Excluded patients with preexisting pacemaker (*n* = 65) at baseline.

**Table 5:** Predictors of PPM.

	Univariate	*p* Value	Multivariate	*p* Value
**Predictors**				
Age (>84 years)	1.23 (0.82, 1.83)	0.316		
CAD (%)	1.08 (0.73, 1.60)	0.690		
Annulus area (>3.38 mm^2^)	*0.22 (0.12, 0.40)*	*** <0.001 ***	*excluded ^1^*	
Cover index, STJ ( >−0.19%)	2.95 (1.88, 4.63)	**<0.001**	3.26 (2.03–5.23)	<0.001
Cover index, Perimeter (>4.74%)	0.81 (0.55, 1.20)	0.302		
BMI (>22.77 kg/m^2^)	*2.90 (1.59, 5.30)*	*** <0.001 ***	*excluded ^1^*	
EuroScore II (>2.49)	1.27 (0.82, 1.96)	0.278		
Depth LCC (>5.7 mm)	1.64 (1.09, 2.47)	**0.017**	2.25 (1.44–3.53)	**<0.001**
LVOT calcification	1.18 (0.40, 3.45)	0.767		
Mean transaortic pressure (mmHg)	2.12 (1.33, 3.37)	**0.002**	1.49 (0.85–2.61)	0.157
Severe calcification (>1690 HU)	2.01 (1.35, 3.00)	**<0.001**	2.07 (1.29–3.33)	**0.002**
Post dilatation	0.81 (0.51, 1.27)	0.351		
Ejection fraction (<55%)	1.33 (0.73, 2.43)	0.358		

Abbreviation: CAD = coronary artery disease; STJ = sinutubular junction; LCC = left coronary cusp; LVOT = left ventricular outflow tract. ^1^ Excluded from the analysis because they define the target variable.

## Data Availability

Data is contained within the article.

## Abbreviations

Ca-density	calcium density
CI	cover index
LVOT	left ventricular outflow tract
MDCT	multidetector computed tomography
PVL	paravalvular leakage
STJ	sinotubular junction
TAVR	transcatheter aortic valve replacement
THV	transcatheter heart valve
PPM	Prosthesis–patient mismatch
iAVA	indexed aortic valve area
